# An inhibitor of complement C5 provides structural insights into activation

**DOI:** 10.1073/pnas.1909973116

**Published:** 2019-12-23

**Authors:** Martin P. Reichhardt, Steven Johnson, Terence Tang, Thomas Morgan, Nchimunya Tebeka, Niko Popitsch, Justin C. Deme, Matthijs M. Jore, Susan M. Lea

**Affiliations:** ^a^Sir William Dunn School of Pathology, University of Oxford, OX1 3RE Oxford, United Kingdom;; ^b^Wellcome Centre for Human Genetics, University of Oxford, OX3 7BN Oxford, United Kingdom;; ^c^Central Oxford Structural Molecular Imaging Centre, University of Oxford, OX1 3RE Oxford, United Kingdom

**Keywords:** complement regulation, innate immunity, inhibitor, single-particle cryo-EM, X-ray crystallography

## Abstract

The complement system is a crucial part of innate immune defenses against invading pathogens. The blood-meal of the tick *Rhipicephalus pulchellus* lasts for days, and the tick must therefore rely on inhibitors to counter complement activation. We have identified a class of inhibitors from tick saliva, the CirpT family, and generated detailed structural data revealing their mechanism of action. We show direct binding of a CirpT to complement C5 and have determined the structure of the C5–CirpT complex by cryoelectron microscopy. This reveals an interaction with the peripheral macro globulin domain 4 (C5_MG4) of C5. To achieve higher resolution detail, the structure of the C5_MG4–CirpT complex was solved by X-ray crystallography (at 2.7 Å). We thus present the fold of the CirpT protein family, and provide detailed mechanistic insights into its inhibitory function. Analysis of the binding interface reveals a mechanism of C5 inhibition, and provides information to expand our biological understanding of the activation of C5, and thus the terminal complement pathway.

## Significance

The complement system is a crucial antimicrobial system in the human body. However, controlling its regulation is essential, and failure to do so is implicated in a number of clinical inflammatory pathologies leading to great interest in therapeutic complement inhibition. We have identified and characterized a class of complement inhibitors from biting ticks. Utilizing both cryoelectron microscopy and X-ray crystallography we provide a comprehensive understanding of their mechanism of inhibition at the level of the terminal pathway of complement. We present a high-resolution cryoelectron microscopy structure of complement C5, the molecule targeted by the major therapeutic Eculizumab. In addition, we reveal the fold of the CirpT family of tick inhibitors and their unique mode of inhibition.

The bloodmeals of some ticks may last several days, providing ample time for their target to mount a full immune response against the tick during their feeding, and ticks within the same colony will rebite individuals multiple times, further enhancing immune responses and exposing the tick to their deleterious effects. To survive, ticks have evolved potent inhibitors of mammalian immunity and inflammation. Tick saliva thus represents an interesting target for the discovery of novel immune system modulators, in particular, inhibitors of the very early initiators of inflammation, such as the complement system.

The complement system plays a major role in targeting the innate immune defense system, and is primarily involved in antimicrobial defense, clearance of apoptotic cells and immune complexes, and finally immune regulation (1, 2). Activation may be initiated by target-binding of pattern recognition molecules, such as C1q (classic pathway), mannose binding lectin, ficolins, or collectins (lectin pathway) (3). In addition, the alternative pathway may autoactivate, including targeting of endogenous surfaces, where inhibitor molecules then terminate further activation (4, 5). The 3 pathways all converge at the activating cleavage of C3 into C3a and C3b, and the subsequent activating cleavage of C5 into C5a and C5b. C3a and C5a are potent anaphylatoxins acting as soluble inflammatory mediators, while C3b and C5b are deposited on target surfaces. C3b and its inactivated form iC3b function as opsonins for phagocytes, while C5b initiates the terminal pathway by assembly of the pore-forming membrane attack complex (MAC, C5b-C9) (6).

With the ability of complement to target self-surfaces and induce potent inflammatory responses, the appropriate regulation of complement is essential. Insufficient control of activation is associated with excessive inflammation, tissue damage, and autoimmunity (7, 8). Inhibiting activation of C5, and thus the generation of C5a and MAC, has shown great therapeutic benefit in complement-driven inflammatory diseases, such as atypical hemolytic uremic syndrome (aHUS) and paroxysmal nocturnal hemoglobinuria (PNH) (9, 10). The specific targeting of C5 limits the potency of complement activation, while still allowing the effects of the upstream opsonization by C4b and C3b, as well as the immune signaling mediated through C3a. The treatment consists of an anti-C5 antibody that blocks convertase-binding (Eculizumab) (11, 12). However, this antibody is 1 of the most expensive drugs in the world, and further therapeutic developments are therefore important. Novel inhibitors, such as the tick protein OmCI (Coversin), the RNAi Aln-CC5, as well as 2 anti-C5 minibodies (Mubodina and Ergidina) are currently undergoing clinical trials, but a better mechanistic understanding of the activation of C5 is necessary to fundamentally improve the therapies for diseases associated with uncontrolled complement activation (13, 14).

To address the need for a more detailed understanding of the mechanisms of C5 activation, we have identified and characterized a family of C5 inhibitors from tick saliva, hereafter named the CirpT (complement inhibitor from *Rhipicephalus pulchellus* of the terminal pathway) family. We show that the CirpT family of inhibitors functions by targeting a novel site on C5, and thus provide essential mechanistic evidence for our understanding of C5 activation. We present the cryoelectron microscopy (cryo-EM) structure at 3.5 Å of human C5 in complex with the previously characterized tick inhibitors OmCI and RaCI, as well as a member of the CirpT family (CirpT1). Based on the cryo-EM structure, we then solved the 2.7-Å crystal structure of CirpT1 bound to macroglobulin domain 4 of C5 (C5_MG4). Analysis of the specific binding interactions between C5 and CirpT1 suggests that the CirpT family functions by direct steric blocking of the docking of C5 onto the C5-convertase, and our data thus provide support for previous models of C5 activation, which include convertase binding through C5 domains MG4, MG5, and MG7.

## Results

### Identification of a Complement Inhibitor.

**Fig. 1.**
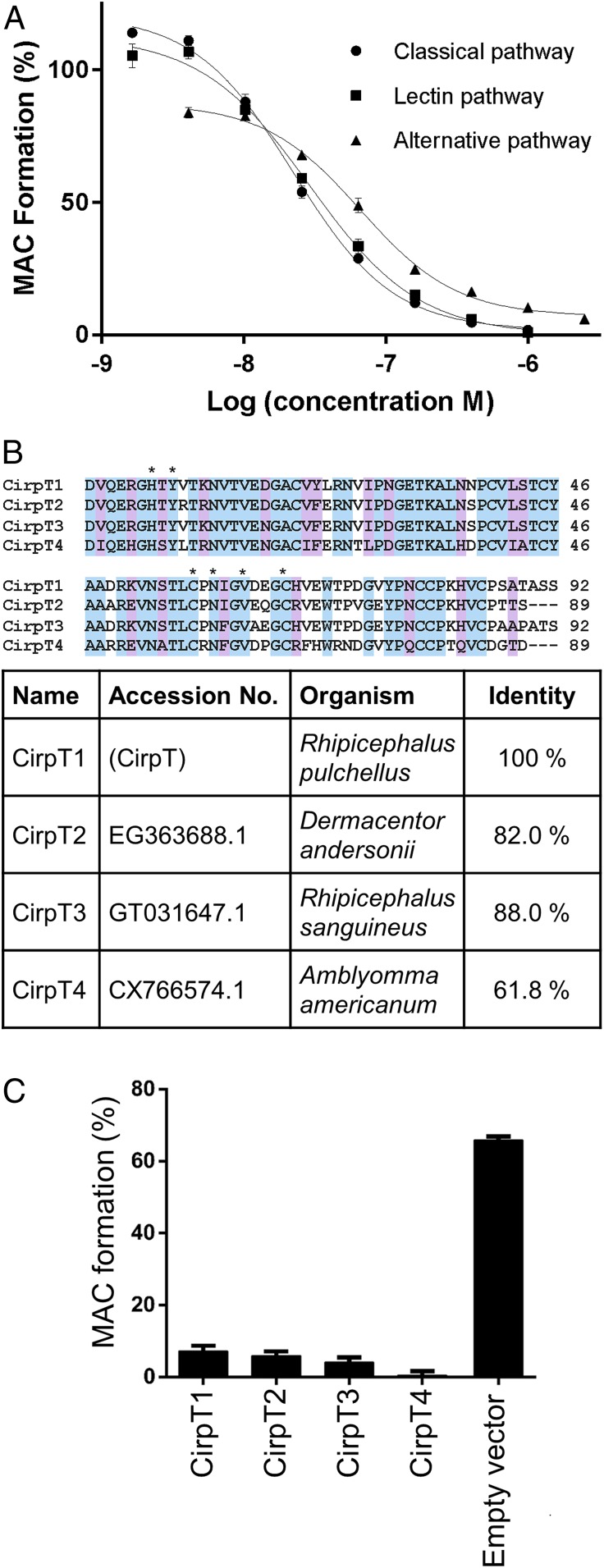
A class of tick proteins (CirpTs) inhibit complement activation. Sequential fractionation of tick salivary glands revealed a family of tick complement inhibitors. Following recombinant expression, purified tick proteins and culture supernatants from S2-insect cells overexpressing tick molecules were utilized to determine the potential for complement inhibition. (*A*) In the commercial Wieslab assay, inhibition of pathway-specific complement activation was tested. Addition of CirpT1 purified from *E. coli* reveal a dose-dependent inhibition of MAC deposition in all 3 pathways. The lines are showing nonlinear fits. Error bars: SEM, *n* = 3. (*B*) Clustal Omega (European Molecular Biology Laboratory-European Bioinformatics Institute) sequence alignment of CirpT1-4. The native signal peptides are omitted from this alignment. Coloring based on sequence identity. Stars (*) denote residues relevant for protein interaction (see below). Sequence identity to CirpT1 is indicated in the table. (*C*) In the commercial Wieslab Alternative pathway assay, ELISA-wells are coated with LPS and will lead to complement activation and deposition of the MAC. Addition of culture-supernatants from insect cells expressing CirpT1-4 inhibits MAC-formation. No inhibition is seen with supernatants from cells transfected with an empty vector, error bars: SEM, *n* = 3.

To identify novel complement inhibitors from tick saliva, salivary glands from the tick *R. pulchellus* were extracted, homogenized, and fractionated utilizing a series of chromatographic methods. In each step, flow-through and elution fractions were tested for their ability to inhibit MAC deposition in a standard complement activation assay using human serum (Wieslab), and the active fractions were further fractionated (*SI Appendix*, Fig. S1 *A*–*C*). The proteins in the final active fraction were analyzed by electrospray ionization-MS/MS following trypsin digest. Initial analysis against a peptide database generated from the published transcriptome database (15) gave no relevant hits. Therefore, a tick sialome cDNA library was assembled from raw sequence data from the Sequence Read Archive (accession no. PRJNA170743, National Center for Biotechnology Information, NCBI). Using a peptide library generated from this transcriptome, we obtained a list of 44 protein hits, 12 of which contained a predicted N-terminal signal peptide, and were not previously found to be expressed in the published database (*SI Appendix*, Table S1). These were expressed recombinantly in *Drosophila melanogaster* S2 cells and the culture supernatants tested for complement inhibitory activity (*SI Appendix*, Fig. S1*D*). One protein, subsequently termed CirpT, was shown to inhibit MAC assembly regardless of the initiation pathway of complement (Fig. 1*A*). To identify potential biologically relevant homologs, the CirpT sequence was used to query the expressed sequence tag database (NCBI) as well as in-house *Rhipicephalus appendiculatus* and *R. pulchellus* sialomes. This search revealed that the protein is highly conserved among ticks, with homologs found throughout the genii *Rhipicephalus* and *Amblyomma*, as well as in the species *Dermacentor andersonii* and *Hyalomma marginatum* (Fig. 1*B*). The proteins identified fall into 4 distinct clusters, with sequence identity varying between 62.6% and 88.3% within the clusters. For further investigation of the mechanisms of these types of complement inhibitors, a member from each cluster (hereafter named CirpT1-4) was expressed in *D. melanogaster* S2 cells and all were shown to inhibit complement activation (Fig. 1*C*). For subsequent studies, all 4 homologs were expressed in *Escherichia coli* SHuffle cells with an N-terminal His-tag and purified using Ni-chelate chromatography, ion-exchange, and size-exclusion chromatography (SEC).

**Fig. 2.**
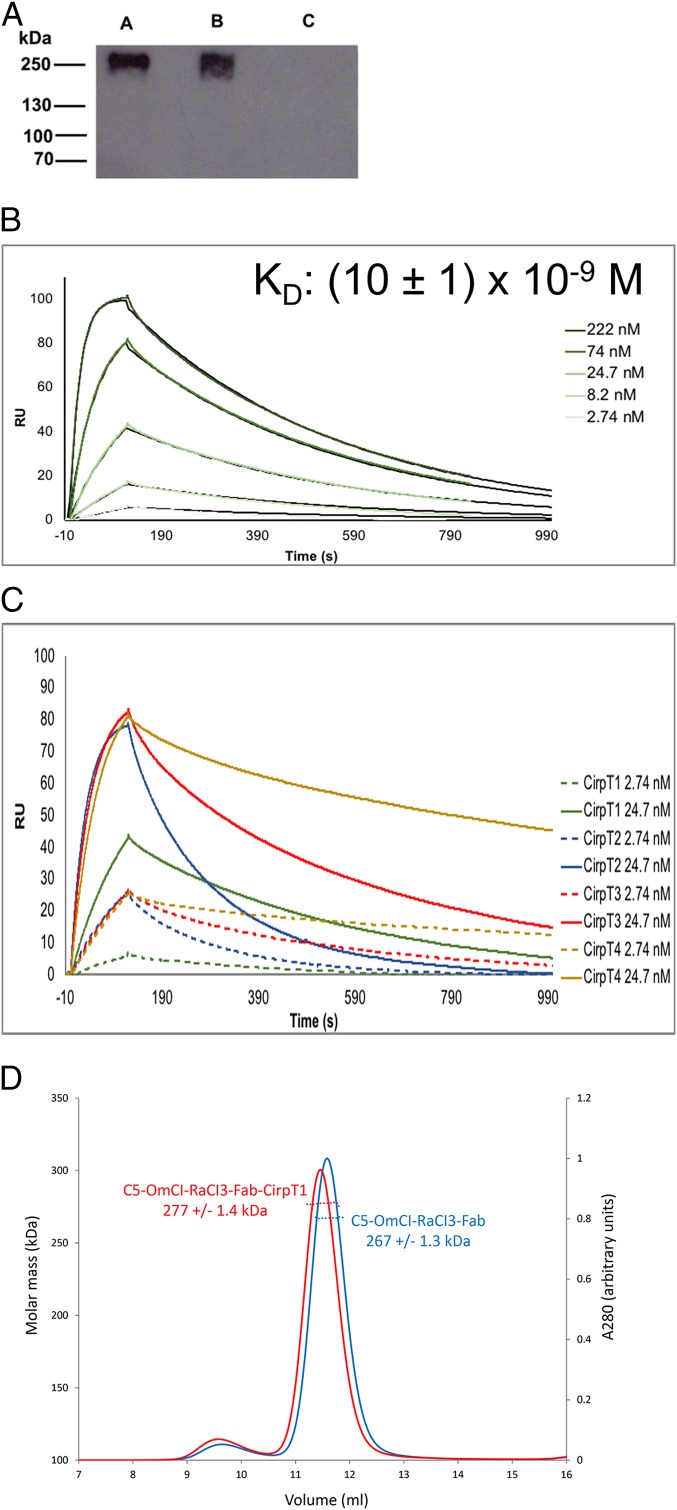
CirpT1 binds C5 through a binding site not overlapping with known inhibitors. (*A*) Western blotting of serum pull-down; 0.5 mg/mL purified CirpT1 was immobilized on NHS-activated magnetic beads (Pierce, Thermofisher Scientific) and incubated with human serum. Eluted proteins were separated by 4 to 12% gradient SDS/PAGE and visualized by Western blotting using a polyclonal anti-C5 antibody. Pull-down lanes A: CirpT1, B: OmCI, C: beads only. (*B*) SPR performed with purified C5 coupled to a CM5 chip by amine-coupling. CirpT1 was flown over in a concentration series from 2.74 to 222 nM, as indicated. Shown are representative curves of CirpT1 flown over surface with 3 different levels of coupled C5. An approximate dissociation constant was calculated by kinetic curve-fitting using the BiaEvaluation software package (*n* = 1). (*C*) SPR of CirpT1-4. All CirpTs clearly bind. The binding curves of CirpT2-4 could not be reliably fit, but our data show even tighter interactions for CirpT2-4 than CirpT1. (*D*) SEC-MALS traces of purified C5 complexed with the inhibitory molecules OmCI, RaCI3, CirpT1, and the Fab-fragment of the commercial antibody Eculizumab. Binding of CirpT1 does not compete with any of the other inhibitory molecules, revealing a mechanism of inhibition.

To pinpoint the specific ligand of CirpT, a pull-down assay from human serum was performed utilizing CirpT1 that was covalently coupled to beads. This identified C5 as the target of CirpT inhibition (Fig. 2*A*). Following this, binding of each CirpT1-4 to C5 was assayed by surface plasmon resonance (SPR). C5 was coupled onto a CM5 chip surface by standard amine-coupling, and CirpT1-4 were flown over in varying concentrations. Analysis of the binding curves for CirpT1 using a 1:1 kinetic model (Fig. 2*B*) yielded a dissociation constant of 10 × 10^−9^ M (averaged over 3 single experiments with varying amounts of C5 coupled to the chip surface). Attempts to produce data with reliable fits for CirpT2-4 were unsuccessful, in part due to long dissociation times and an inability to regenerate the binding surface without denaturing the C5. However, the magnitude of binding signal at comparable concentrations demonstrates that they likely bind tighter than CirpT1, with CirpT4 displaying an especially slow dissociation rate (Fig. 2*C*). The *K*_D_ of all CirpT-C5 affinities are thus in the range of 10 nM or tighter.

It was previously demonstrated that other tick inhibitors targeting C5, OmCI, RaCI, SSL7, as well as the Fab-fragment of the commercially available C5-inhibitory antibody, Eculizumab, have different binding sites on C5 (11, 16). We next sought to compare the binding mechanism of the CirpTs to the previously known modes of inhibition. C5, OmCI, and RaCI were purified and complexed with the Eculizumab Fab-fragment and the mass was determined by SEC-multiangle light scattering (MALS). A 10-kDa increase in the mass of the complex was observed when CirpT1 was added to the quaternary complex, demonstrating that the binding site for CirpT does not overlap with any previously known inhibitors (Fig. 2*D*). The mass increase was consistent with CirpT binding as a monomer. CirpT thus has a mechanism for inhibition of complement C5 activation which has not previously been described.

### Cryo-EM Structure of the C5–OmCI–RaCI–CirpT1 Complex.

**Fig. 3.**
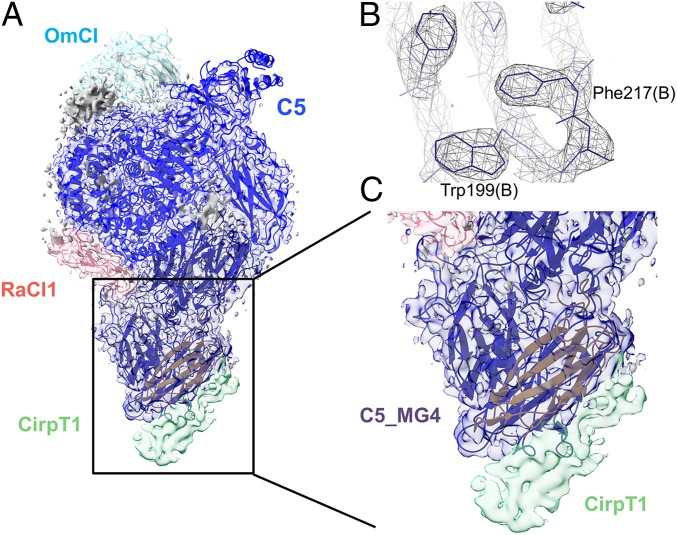
Cryo-EM structure of the C5–OmCI–RaCI–CirpT1 complex. (*A*) Side view of the density map with C5 (blue), OmCI (light blue) and RaCI1 (red) structures built in. Residual density (green) was observed attached to the macroglobulin domain 4 (C5_MG4, gold). This residual density was attributed to CirpT1. (*B*) Example of higher-resolution data in the density map, allowing the placement of amino acid sidechains in the density. The map represented here has been postprocessed with B-factor sharpening. (*C*) Zoom of C5_MG4 with the CirpT1 density clearly visible. The map represented here is filtered specifically to provide best detailed information for CirpT1.

To characterize the mechanism of C5 inhibition, we next identified the binding site of CirpT1 on C5 by cryo-EM. As OmCI and RaCI lock C5 into a less-flexible conformation, our approach targeted the full C5–OmCI–RaCI–CirpT1 complex. Purified OmCI–C5 was incubated with a 2-fold molar excess of RaCI and CirpT1, and the complex was purified by SEC. The complex was imaged on a Titan Krios and a 3D reconstruction was generated at a nominal overall resolution of 3.5 Å from 118,365 particles (Fig. 3*A*, Table 1, and *SI Appendix*, Fig. S2). The volume generated allowed us to dock and refine the previously determined crystal structure of the C5, OmCI, and RaCI1 complex. As observed in the ternary complex crystal structures, local resolution varied across C5 within the complex, with the C345c domain being the least well-ordered part of the complex (5 to 6 Å). However, domains buried within the core of the C5 demonstrated features consistent with the experimentally determined local resolution of 3.35 Å, including detailed sidechain density (Fig. 3*B*). By comparing the expected density from the crystal structure of the C5–OmCI–RaCI complex to our newly generated map, we identified an extra density in the lower right corner of the complex, which we attributed to CirpT1 (Fig. 3*C*). The local resolution in this area was worse than for the overall complex (4 to 5 Å), indicating a higher level of flexibility. The resolution of the map corresponding to CirpT1 was insufficient to build a de novo atomic model of CirpT1. However, the residual density suggested that the main interaction between C5 and CirpT1 was mediated through binding to C5_MG4 (C5 residues L349 to S458).

**Table 1.** Cryo-EM data collection, refinement, and validation statistics

	C5–OmCI–RaCI1–CirpT1 (EMD ID code 4983) (PDB ID code 6RQJ)
Data collection and processing	
Magnification	165,000
Electron exposure (e–/Å^2^)	48 (K2)
Voltage (V)	300
Pixel size (Å)	0.822
Symmetry	C1
Initial particle images (no.)	502,640
Final particle images (no.)	118,634
Map resolution (Å)	3.53
FSC threshold	0.143
Refinement	
Initial model used (PDB code)	5HCE/6RPT
Model resolution (Å)	3.53
FSC threshold	0.143
Map sharpening *B* factor (Å^2^)	−108
Model composition	
Nonhydrogen atoms	14,971
Protein residues	1,899
Ligands	2
*B* factors (Å^2^)	
Protein	50
Ligand	78
Rmsd	
Bond lengths (Å)	0.006
Bond angles (°)	0.616
Validation	
MolProbity score	2.0
Clashscore	10.9
Poor rotamers (%)	0.06
Ramachandran plot	
Favored (%)	92.9
Allowed (%)	6.9
Disallowed (%)	0.2

### Crystal Structure of the C5_MG4–CirpT1 Complex.

**Fig. 4.**
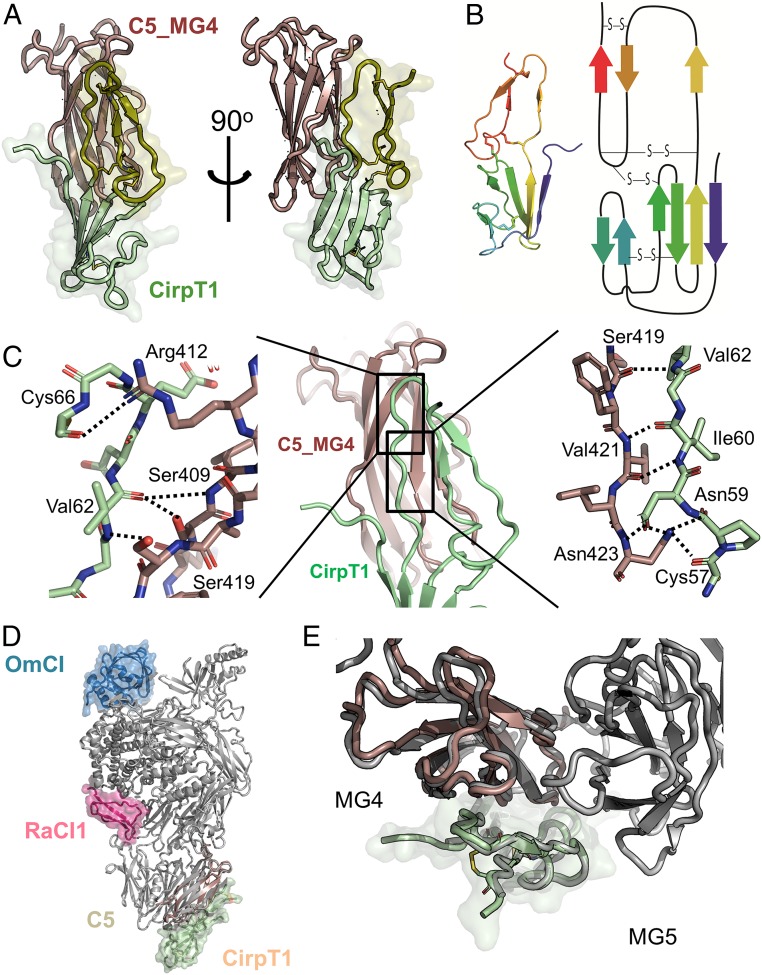
Crystal structure of the C5_MG4–CirpT1 complex at 2.7 Å. (*A*) Front and side views of the crystal structure of the C5_MG4–CirpT1 complex. C5_MG4 shown in brown, CirpT1 shown in green shades. Disulphide bridges are shown in yellow. (*B*) Topology diagram of CirpT1 colored as a rainbow from the N terminus (blue) to C terminus (red). (*C*) Front view cartoon of CirpT1 (green) binding to C5_MG4 (brown) with close up of 2 interaction surfaces. Interacting amino acids are displayed as sticks and hydrogen bonds are highlighted in dashed lines. (*D*) Overlay of the C5_MG4–CirpT1 structure with the full C5–OmCI–RaCI1–CirpT1 complex reveals that CirpT1 sits in the density observed from the cryo-EM (front view). C5 shown in gray, RaCI1 in red, and OmCI in blue. (*E*) A closer investigation of the placement of CirpT1 shows it sits between C5_MG4 and C5_MG5 (top view). Although the major interaction is with C5_MG4, and this is sufficient for binding, the structural overlay shows interaction with the C5_MG5 as well.

To understand the interaction between CirpT1 and C5 in greater detail, a complex of CirpT1 and C5_MG4 was crystallized. To this end, the C5_MG4 domain was cloned with an N-terminal His-tag, expressed in *E. coli*, and purified by Ni-chelate and SEC. To confirm our previous observation of a direct interaction between CirpT1 and C5_MG4, binding was tested by coelution on a size-exclusion column. This yielded a C5_MG4–CirpT1 complex, which was purified, concentrated, and crystallized. X-ray diffraction data to 2.7 Å were collected at the Diamond Light Source (beamline I03). The structure of the C5_MG4–CirpT1 complex was solved by molecular replacement with the isolated C5_MG4 extracted from the C5–OmCI–RacI complex. Initial phases from the partial molecular replacement solution produced a map into which a model of CirpT1 was built de novo, and the complex refined to give the model described in Fig. 4*A* and Table 2.

**Table 2.** X-ray data collection and refinement statistics

	C5_MG4–CirpT1(PDB ID code 6RPT)
Data collection	
Space group	P 1 21 1
Cell dimensions	
*a*, *b*, *c* (Å)	86.83, 56.95, 90.07
α, β, γ (°)	90.00, 113.01, 90.00
Resolution (Å)	82.90–2.52 (2.56–2.52)*
*R*_merge_	0.266 (2.469)
*I*/σ*I*	3.8 (0.9)
Completeness (%)	99.8 (98.5)
Redundancy	6.5 (6.7)
Refinement	
Resolution (Å)	82.94–2.7 (2.81–2.7)
No. reflections	22,552 (2243)
*R*_work_/*R*_free_	0.226/0.272 (0.306/0.343)
No. atoms	
Protein	4,518
Ligand/ion	30
Water	110
*B*-factors	
Protein	51.83
Ligand/ion	64.88
Water	34.68
Rmsd	
Bond lengths (Å)	0.002
Bond angles (°)	0.577

* Values shown in parentheses are for highest-resolution shell.

CirpT1 is made up of 2 domains: A bulky N-terminal domain and a flat looped C-terminal domain (Fig. 4*B*). A Fold and Function Assignment Service (FFAS) search (17) of the CirpT1 sequence showed overall similarities to the von Willebrand (VW) factor type C (VWC) domain family (18), with 4 conserved cysteine bridges (score: −22.2, 26% identity). The N terminus of CirpT1 extends further, however, and engages in a 4-stranded β-sheet. Using the Dali server (19), searches for either the bulky N-terminal domain of CirpT1 (amino acids 2 to 56) or the full CirpT1 (amino acids 2 to 87) identified the porcine β-microseminoprotein (MSMB, PDB ID code 2IZ4-a) as the closest structural homolog (rmsd of 1.8 Å with 46 of 91 residues aligned for the N-terminal domain, and rmsd of 4.2 Å with 72 of 91 residues aligned for the full CirpT1). In both CirpT1 and MSMB, the N termini form a Greek key motif with 4 antiparallel strands, with an extended loop between the 1st and 4th strands. This extended loop folds back under the β-sheet, and is locked into this confirmation by a disulfide-bridge to the 3rd β-strand in the sheet. The C-terminal domain of CirpT1 corresponds more closely to the VWC fold, but a Dali search for this domain alone did not yield any hits. The orientation of the N-terminal and C-terminal domains in relation to each other differ from known homologs, an observation consistent with flexibility reported in the hinge-region of the VW folds (18).

A detailed analysis of the binding interface was carried out using PDB-ePISA (20). This revealed an extensive interface that encompasses most of the C-terminal domain of the CirpT1 (Fig. 4*C*), with additional contributions from the N-terminal strand and 2 residues at the tip of the extended loop in the N-terminal domain (Tyr23 and Leu24). The interface is predominantly hydrophobic in nature, with His7, Tyr23, and Asn59 on CirpT1 contributing sidechain hydrogen bonds (to Asp405, Ser426, and Asn423, respectively). Overlay of the C5_MG4–CirpT1 structure onto the C5–OmCI–RaCI–CirpT1 structure revealed minor clashes between CirpT1 and the neighboring C5_MG5 domain that were resolved by a small rigid body movement of the CirpT1 into the cryo-EM density (Fig. 4 *D* and *E*). This placement of CirpT1 in the context of intact C5 revealed additional contacts between CirpT1 and C5_MG5, including a hydrophobic cleft between C5_MG4 and C5_MG5 that Trp70 and Pro72 of CirpT1 pack into. Analysis of the sequence conservation of the CirpTs (Fig. 1*B*) revealed that the residues involved in binding C5 are highly conserved, with residues His7, Tyr9, Cys57, Asn59, Val62, and Cys66 conserved among all 4 CirpTs examined. Of note, the stretch of residues from Cys66-Glu69 in CirpT1 extend over an extension of the hydrophobic crevice between C5_MG4 and C5_MG5, but leaving a hydrophobic hole. CirpT4, which appeared to have the slowest dissociation rate in the SPR studies, has a Val68Phe substitution that would fill this cavity, further stabilizing the complex.

### Species Specificity of CirpT.

**Fig. 5.**
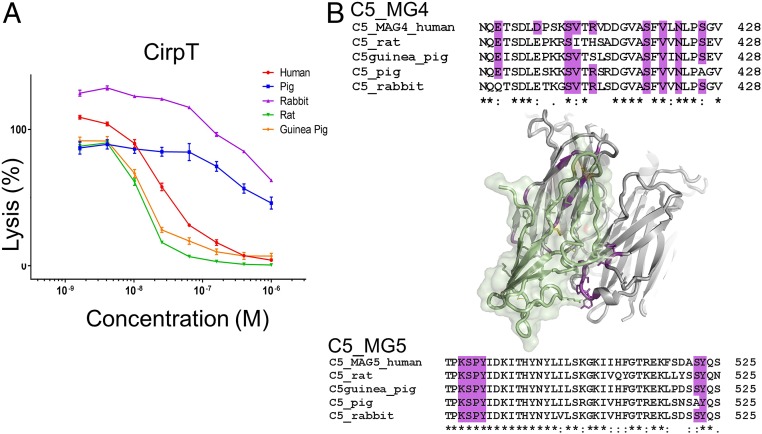
Species specificity of CirpT. (*A*) Serum complement-mediated lysis of red blood cells was assayed with increasing concentrations of CirpT1 inhibitor. A dose-dependent inhibition was observed for all tested species: Human, pig, rabbit, rat, and guinea pig. Lysis in animal sera were normalized against the base-line lysis of human serum without inhibitor added. Error bars: SEM, *n* = 3. (*B*) Sequence alignment of C5_MG4 and C5_MG5 of tested species in the PISA-predicted CirpT binding interface. This reveals a high level of sequence conservation, thus explaining the potent inhibitory effect of CirpT across species (residues involved in binding are highlighted in purple). Sequence identity is denoted by: * (100 %), and : (strongly similar chemical properties).

Ticks feed on a wide variety of mammalian species, and they are therefore required to have potent inhibitors of complement from multiple species. We tested the ability of CirpT1 to inhibit complement mediated red blood cell lysis by serum from rat, pig, guinea pig, and rabbit (Fig. 5*A*). We observed inhibition in all species investigated, although inhibition by rabbit and pig serum was less potent. Mapping C5 sequence conservation in the binding interface demonstrated that the key residues involved in binding CirpT1 are highly conserved across these species (Fig. 5*B*), explaining this broad spectrum of activity. Specifically, the C5-residues Glu398, Ser407, Val408, Ser417, Val419, Asn421, and Ser424 are all highly conserved with only single substitutions in particular species (rabbit Glu398Gln, rat Val408Ile, and pig Ser417Ala). The salt-bridge–forming Asp403 in human is substituted with a glutamic acid in all other species. Arg410 is replaced by a histidine and a serine in rat and guinea pig, respectively. In addition, the potential binding interface to C5_MG5 is also highly conserved. LysSerProTyr489-492 and Tyr523 are conserved in all tested species, while Ser522 is found in all species except the pig (substitution to Ala522).

### Mechanism of Complement Inhibition.

**Fig. 6.**
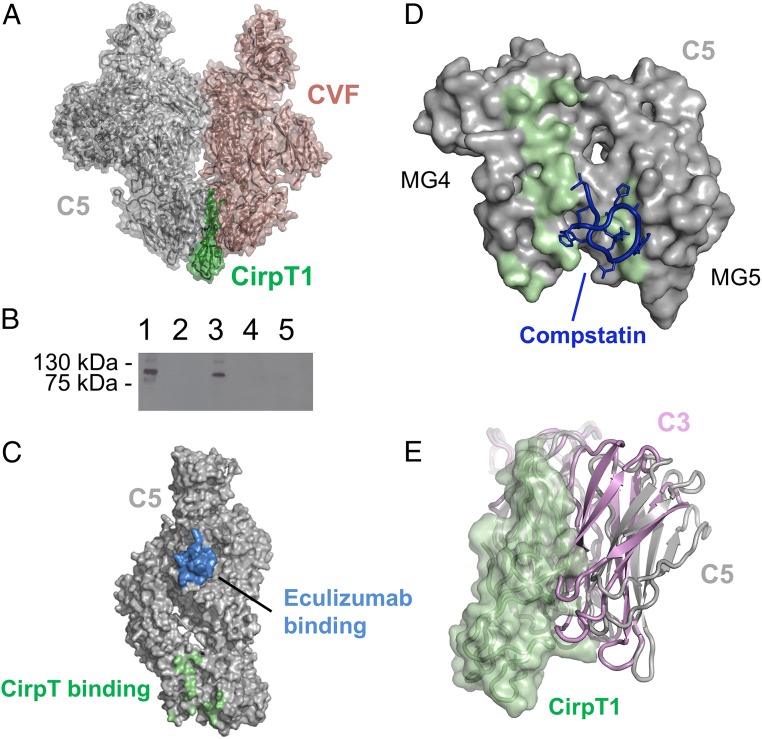
Mechanism of inhibition. (*A*) CirpT (green) overlaid with the complex between C5 (gray) and CVF (brown). CirpT1 sits right at the C5–CVF (PDB ID code 3PVM) binding interface. (*B*) Western blotting (αC5, reducing conditions) of elutions from C3b-coated magnetic beads. Lane 1: Purified C5. Lane 2: C5 + empty beads. Lane 3: C5 + C3b-coated beads. Lane 4: C5 + CirpT1 + empty beads. Lane 5: C5 + CirpT1 + C3b-coated beads. (*C*) Surface representation of C5. Residues of MG7 indicated in binding to the clinical antibody Eculizumab are highlighted in blue. Residues of MG4 and MG5 indicated in binding to CirpT are highlighted in green. (*D*) Surface representation of C5 MG4 and MG5 with the CirpT binding site highlighted in green. The C3 inhibitor Compstatin is modeled as sticks in blue, based on the location of its binding site on the equivalent domains of C3. (*E*) Sideview of the overlay of the CirpT1 (green) with C5 (gray) and C3 (pink, PDB ID code 2A73). The domain organization of C3_MG5 in relation to C3_MG4 shows a much closer conformation as compared to C5. C3_MG5 is thus likely to provide steric hindrance of the CirpT inhibitor, thus explaining the specific C5-targeting of the tick molecule.

Current models propose that C5 is activated by docking to C3b, complexed with either of the activating proteases C2a or Bb, resulting in C5 cleavage into C5a and C5b. A key interaction between C5 and C3b, based on structures of C5 bound to the C3b homolog cobra venom factor (CVF), is thought to involve the packing of C5_MG4 and C5_MG5 against the C3b-containing convertase (16). Mapping of the residues involved in CVF binding onto the C5 structure reveals extensive overlap with the CirpT binding site and superposition of the C5–OmCI–RaCI–CirpT and C5–CVF structures confirms major steric clashes between CirpT and CVF (Fig. 6*A*). In order to test the hypothesis that CirpT would therefore inhibit C3b binding to C5, we analyzed the binding of C5 to beads coated with C3b, thus mimicking the high critical surface concentration of C3b required for C5 conversion (21). Purified C3 was activated by trypsin-cleavage and the resulting C3b was biotinylated and coupled to streptavidin magnetic beads. Purified C5 specifically bound to the C3b-coated beads and this interaction was prevented by preincubation of the C5 with CirpT1, thus supporting the steric inhibition model of complement inhibition (Fig. 6*B*).

## Discussion

In an effort to understand the mechanisms underlying complement activation, we utilized the complement inhibitory properties of tick saliva. We herein describe a class of tick inhibitory molecules targeting the terminal pathway of complement: CirpT. Homologous sequences were identified in several tick species and genii, and fell into 4 distinct clusters, CirpT1-4. They all share a high degree of sequence identity with CirpT1 (62.6 to 88.3%), and all inhibit complement activation through binding to C5 with nanomolar affinities. To understand this mechanism of complement inhibition, we generated a cryo-EM structure of C5 complexed with the tick inhibitors OmCI, RaCI, and CirpT1, simultaneously verifying the previously determined binding mechanisms of OmCI and RaCI, and revealing the binding site of CirpT. This facilitated a targeted crystallographic approach that produced a 2.7 Å structure of the C5_MG4 domain in complex with CirpT1.

The CirpT family adopts an extended 2-domain fold stabilized by multiple disulphide bonds. An extended strand connects the compact β-sandwich of the N-terminal domain to a flatter C-terminal domain with less canonical secondary structure. This flat domain is further connected to the bulky domain by a disulfide bridge. The limited connection between the 2 subdomains allows for a flexibility of this “hinge-region,” which can likely account for the small variation between the crystal structure and the cryo-EM structure of CirpT1. Flexibility between the 2 subdomains is consistent with similar flexibility reported for the VW folds (18).

Cleavage of the homologous molecules C3 and C5 is carried out by mechanistically similar convertases. The C3 convertase consists of a docking-molecule (C3b or C4b) associated with an activated serine protease (either factor Bb or C2a, respectively). The C3 convertases C4b2a (classic/lectin pathways) and C3bBb (alternative pathway) generate surface-associated C3b (hereafter termed C3b′), which subsequently associates with additional factor Bb, and thus provides a potent amplification loop. Following the deposition of critically high concentrations of C3b′ on a surface, a shift in convertase activity is created, permitting C5 as a substrate (21–26). Current models of C5 activation include docking of C5 to C3b, complexed with either of the activating proteases C2a or Bb (11, 26). Previous work utilizing the C3b homolog CVF has provided structural information of how this docking may occur (16). Structural data of C5 complexed with CVF (PDB ID code 3PVM) show that C5 domains MG4, MG5, and MG7 are involved in this interaction. The main binding interface is found with surface residues of MG4 and MG5. The C5 MG4 and MG5 residues Ser419–Pro425, Thr470–Ile485, and Asp520–Asn527 are all located in proximity to the CVF residues Ser386–Thr389, Ile399–Leu404, Thr450–Lys467, and Arg498–Asn507 (CVF domains MG4 and MG5). The CirpT1 binding interface with C5 directly overlaps with this binding interface. We therefore propose that the CirpT mechanism of inhibition is through direct steric hindrance of C5-docking to its convertase. This is substantiated by the observed CirpT1-mediated inhibition of C5 binding to surface-coated C3b. Our structural data thus support the CVF-generated model of C5-convertase docking, and provides further evidence for this being a crucial part of the mechanism of C5-activation in humans (Fig. 6 *A* and *B*). C5-convertase activity is dependent on a high density of surface-associated C3b (21, 26). Combined with structural knowledge of C5-inhibitors such as OmCI and RaCI, a hypothesis suggesting 2 distinct binding interfaces between C5 and C3b has emerged (11, 21, 26). The data presented here indicate, that the binding-interface utilizing C5_MG4 is essential for sufficient binding of C5 to C3b.

In a number of complement-driven inflammatory diseases, such as aHUS, PNH, and neuromyelitis optica, 2 antibody-based C5 inhibitors targeting the same epitope on C5_MG7 currently represent the only approved treatments (14, 27). However, these therapeutics are associated with substantial costs, and certain population groups have proven nonresponsive due to a polymorphic variation (R885C/H) of the C5 with the antibody binding site (28). The search for novel inhibitors that target the C5 away from MG7 is therefore of great importance. Our present study of CirpT, combined with earlier structural data mapping the Eculizumab binding site (11, 12), demonstrates that both families of inhibitors act by sterically disrupting binding of the substrate to the convertase (Fig. 6*C*). However, while Eculizumab specifically binds C5_MG7, CirpT interacts with C5 domains MG4 and MG5. This makes CirpT a potentially interesting target for the development of new clinical strategies. As the genetic polymorphism of C5 (R885C/H) rendering patients nonresponsive to Eculizumab-treatment reside in the MG7 domain, the specific targeting of MG4 and MG5 by CirpT is particularly relevant for this patient cohort.

Due to the high levels of homology between C3, C5, and their convertases, we compared our proposed mechanism of inhibition to that of a known C3-inhibitor, compstatin (Fig. 6*D*). The structure of compstatin bound to C3c demonstrated that it targets C3 domains MG4 and MG5 (29) and, based on crystal contacts in the C3b structure (30), it has been suggested that compstatin inhibits C3 binding to its convertase in a manner analogous to the mechanism we propose for C5 and CirpT. An overlay of the C3–compstatin complex with our C5–CirpT1 complex confirms that CirpT1 and compstatin block a similar site on the MG4/5 domains of either complement molecule. However, while CirpT1 and compstatin mediate inhibition through very similar mechanisms, no binding or inhibitory effect of CirpT was observed toward C3. Overlay of the structure of C3 (PDB ID code 2A73) with our C5–OmCI–RaCI–CirpT structure reveals that the neighboring C3_MG5 domain packs much closer to C3_MG4, essentially sterically hindering binding of CirpT1 (Fig. 6*E*).

In conclusion, we here present the structural fold of a family of complement inhibitors. By identifying the specific binding site, we provide further mechanistic insight into current models of C5 activation. Combined with the detailed molecular understanding of multiple C5 inhibitors, our mechanistic understanding may allow for future developments of clinically relevant therapeutic strategies.

## Methods

### Fractionation of *R. pulchellus* Salivary Glands.

*R. pulchellus* ticks were reared and 250 salivary glands were dissected according to Tan et al. (15). The gland protein extract was topped up with 25 mM Na_2_HPO_4_/NaH_2_PO_4_, pH 7.0 to 10 mL. The sample was then fractionated by sequential anion exchange, SEC, and reverse-phase hydrophobic interaction chromatography. At each stage, eluted fractions and flow-through from the chromatographic columns were assayed for complement inhibitory activity, and the active fractions were further fractionated. First, protein extract was fractionated by anion-exchange chromatography using a MonoQ 5/50 GL column (GE Healthcare), washed with 10 column volumes (CV) 25 mM Na_2_HPO_4_/NaH_2_PO_4_, pH 7.0, and eluted by a 0 to 0.5 M NaCl gradient over 30 CV in 500-μL fractions. The flow-through was then acidified by addition of 1 μL 10 M HCl and injected onto a Dynamax 300-Å C8 column (Rainin). The sample was eluted with a 0 to 80% ACN gradient in 0.1% TFA over 40 min. Aliquots were lyophilized and resuspended in 500 μL PBS. The active fraction was incubated at 21 °C for 1 h with an equal volume of 3.4 M (NH_4_)_2_SO_4_, pH 7.0, centrifuged (22,000 × *g*, 10 min) and topped up to 0.95 mL with 1.7 M (NH_4_)_2_SO_4_, 100 mM Na_2_HPO_4_/NaH_2_PO_4_, pH 7.0. The sample was loaded onto a 1-mL HiTrap Butyl HP column (GE Healthcare), and washed with 5 CV of 1.7 M (NH_4_)_2_SO_4_, 100 mM Na_2_HPO_4_/NaH_2_PO_4_, pH 7.0. Elution was carried out by a 1.7 to 0.0 M (NH_4_)_2_SO_4_ gradient over 15 CV in 1-mL fractions. All fractions were buffer exchanged to PBS and concentrated.

### Identification of Tick Inhibitors.

Identified protein fractions with complement-inhibitory abilities were digested by Trypsin and analyzed by LC-MS/MS. Samples were topped up to 50 μL with 50 mM TEAB, pH 8.5, reduced with 20 mM TCEP (21 °C, 30 min), alkylated with 50 mM chloroacetamide in the dark (21 °C, 30 min), digested with 0.5 μg of trypsin (37 °C, 16 h), then quenched with 1 μL formic acid. Digested peptides were analyzed by LC-MS/MS over a 30-min gradient using LTQ XL-Orbitrap (Thermo Scientific) at the Central Proteomics Facility (http://www.proteomics.ox.ac.uk, Sir William Dunn School of Pathology, Oxford, United Kingdom). Data were analyzed using the central proteomics facilities pipeline (31) and peptides were identified by searching against the *R, pulchellus* sialome cDNA database (15) and an updated *R. pulchellus* sialome cDNA database from raw sequence data (Sequence Read Archive, NCBI, accession no. PRJNA170743) with Mascot (Matrix Science). Hits were assessed for the presence of a signal peptide with the SignalP 4.1 Server (32) (Copenhagen Business School, Technical University of Denmark), sequence homology to known protein sequences by blastp (NCBI), and structural homology to known protein structures by FFAS (17).

### *R. pulchellus* Sialome cDNA Database Assembly.

We downloaded female and male *R. pulchellus* sequencing data (100-bp paired-end Illumina HiSeq 2000 reads), as published in ref. 15, from the Sequence Read Archive (accession IDs SRX160117 and SRX160070, respectively). We de novo-assembled female (51Mio read pairs) and male (70Mio read pairs) data independently using Bridger (33) with default parameters. Raw reads were then mapped to the assembled female/male CDNA using NextGenMap 0.4.12 (34), enforcing a minimum 95% sequence identify (-i parameter) and sorted read alignments were inspected in the Integrative Genomics Viewer genome browser (35) for quality control purposes.

### Expression and Purification of Recombinant Proteins.

#### Insect cell expression.

Codon-optimized GeneArt strings were cloned into a modified pExpreS2-2 vector (ExpreS2ion Biotechnologies) with an N-terminal His6 tag (pMJ41). The purified plasmid was transformed into S2 cells grown in EX-CELL 420 (Sigma) with 25 μL ExpreS2 Insect-TR 5X (ExpreS2ion Biotechnologies). Selection for stable cell lines (4 mg/mL geneticin; ThermoFisher) and expansion were carried out according to the manufacturer’s instructions.

#### *E. coli* expression.

GeneArt strings were cloned into pETM-14 and transformed into T7 SHuffle cells (CirpT1-4) or BL21 (DE3) cells (C5_MG4); both cell types were from New England Biolabs. Protein expression was carried out in 2× YT broth (with 50 μg/mL kanamycin). Cells were induced with 1 mM isfopropyl-β-d-thiogalactopyranoside (IPTG). The cultures were centrifuged (3,220 × *g*, 10 min) and the cell pellets resuspended and lysed in PBS containing 1 mg/mL DNase and 1 mg/mL lysozyme by homogenization. Expressed proteins were subsequently purified by Ni-chelate chromatography (Qiagen, 5-mL column) and SEC (S75, 16/60, GE Healthcare) in PBS.

### Complement Inhibition Assays.

Red blood cell hemolysis assays and complement ELISAs were carried out as described previously (11). In brief, haemolysis assay was performed with sheep red blood cells (TCS Biosciences) sensitized with anti-sheep red blood cell stroma antibody (cat. no. S1389, Sigma-Aldrich). Fifty microliters of cells (5 × 108 cells/mL) were incubated in an equal volume of diluted serum (1 h, 37 °C, shaking). Cells were pelleted and haemolysis was quantified at A405 nm of supernatant. Cells with serum only used for normalization (100% activity). Final serum dilutions used was as follows: 1/80 (human), 1/40 (rabbit), 1/160 (rat), 1/40 (pig), and 1/640 (guinea pig). Human serum was from healthy volunteers [prepared as previously described (11)]; pig serum was a kind gift from Tom E. Mollnes, Oslo University Hospital, Norway; rat and guinea pig serum were from Complement Technology and rabbit serum was from Pal Freeze. Complement inhibition ELISAs were performed using a Wieslab complement system screen (Euro Diagnostica) following the manufacturer’s instructions, with sample added prior to serum. Contents were mixed by shaking at 600 rpm for 30 s.

### Pull-Down Assay.

For pull-down assay, 0.1 mg/mL of purified protein was immobilized on Pierce NHS-activated magnetic beads (ThermoFisher) following the manufacturers’ instructions. The beads were incubated with 10 mM EDTA and 50 μL serum (21 °C, 30 min). The beads were washed 3 times with 1 mL PBS + 0.05% Tween20, once with 100 μL PBS, and boiled in 50 μL SDS- PAGE loading buffer. The eluted proteins were separated on an SDS polyacrylamide gel and observed by Coomassie staining, silver staining, or Western blotting. For blotting the SDS/PAGE-separated proteins were transferred to a PVDF membrane (Amersham Hybond P0.2 PVDF, 55 GE) by semiwet transfer (Bio-Rad) and blocked for 1 h with 2% milk. Primary antibody (α-C5: 1:80,000, Complement Technology). Secondary antibody (α-goat HRP, Promega, 1:10,000). The blot was developed using ECL Western Blotting Substrate (Promega) and imaged using Amersham Hyperfilm ECL (GE Healthcare).

### Purification of Serum C5 and C5–Inhibitor Complexes.

C5 and C5–inhibitor complexes were purified essentially as described previously (11). In brief, precleared serum was incubated with His-tagged OmCI, and the C5–OmCI complex was purified by Ni-chelate and anion chromatography (Mono Q 10/100 GL column, GE). A 2-fold molar excess was added of CirpT or RaCI inhibitors, or EcuFab, a custom-made Fab fragment prepared following the manufacturer’s framework (Ab00296-10.6, Absolute Antibody), which includes the VL and VH sequences of Eculizumab (European Patent Office: EP0758904 A1). Following this SEC (S20010/30 HR column, GE Healthcare) was used to remove excess inhibitors purify the final complexes (in PBS). SEC-MALS was performed as described.

### SEC-MALS.

For SEC-MALS, 100 μL of protein sample at 1 mg/mL was injected onto an S200 10/300 column (GE Healthcare) equilibrated in PBS. Light scattering and refractive index were measured using a Dawn Heleos-II light scattering detector and an Optilab-TrEX refractive index monitor. Analysis was carried out using ASTRA 6.1.1.17 software assuming a dn/dc value of 0.186 mL/g.

### SPR.

SPR experiments were performed using a Biacore T200 (GE Healthcare). C5 was coupled to CM5 chips by standard amine coupling. CIRpT1-4 was flown over the surface in 0.01 M Hepes pH 7.4, 0.15 M NaCl, 3 mM EDTA, 0.005% (vol/vol) Surfactant P20, at a flow rate of 30 μL/min. The strong interaction was not sufficiently disrupted by either high/low salt (0 to 3 M NaCl) or extreme pH (range 2 to 8 tried) and extended dissociation time (1 h) was therefore used between successive injections. Fits were performed to control (blank channel)-subtracted traces. Data were fitted using a 1:1 Langmuir with mass transfer model. To calculate the affinity of CIRpT1-4 for C5, a series of injections at concentrations spanning ∼3 nM to 2 μM were fit using the BiaEvaluation software.

### Cryo-EM, Image Processing, Model Building, and Refinement.

C5–OmCI–RaCI–CirpT1 (4 μL) in PBS (0.3 mg/mL) was applied to freshly glow-discharged (20 s, 15 mA) carbon-coated 200-mesh Cu grids (Quantifoil, R1.2/1.3). Following incubation for 10 s, excess solution was removed by blotting with a filter paper for 3 s at 100% humidity at 4 °C, and plunge frozen in liquid ethane using a Vitrobot Mark IV (FEI). Data were collected on a Titan Krios G3 (FEI) operating in counting mode at 300 kV with a GIF energy filter (Gatan) and K2 Summit detector (Gatan). Next, 4,440 movies were collected at a sampling of 0.822 Å/pixel, dose rate of 6 e^−^/Å/s over an 8-s exposure for a total dose of 48 e^−^/Å^2^ over 20 fractions. Initial motion correction and dose-weighting were performed with SIMPLE-unblur (36) and contrast transfer functions (CTFs) of the summed micrographs were calculated using CTFFIND4 (37). Dose-weighted micrographs were subjected to picking using SIMPLE (36) fed with the known crystal structure of C5–OmCI–RaCI complex (PBD ID code 5HCE). All subsequent processing was carried out using Relion 3.0-beta-2 (38). Movies were reprocessed using built-in MOTIONCOR2 (39), with 5 × 5 patches and dose-weighting. Picked particles were extracted in a 288 × 288 Å box, totaling 502,640 particles. Reference-free 2D classification was performed and the highest resolution classes selected, leaving 35,707 particles. Three-dimensional classification was then carried out using a low-resolution ab initio model as a reference and the highest resolution class (118,634 particles) was subjected to masked autorefinement. Following Bayesian polishing and CTF refinement, gold-standard Fourier shell correlations using the 0.143 criterion led to global resolution estimates of 3.5 Å. Postprocessing was carried out using a soft mask and a B-factor of −106 Å^2^ was applied. Local resolution estimations were calculated within Relion 3.0. A model of the C5–OmCI–RaCI1 complex (PDB ID code 5HCE) was fit into the map using the program COOT (40) and refined using the Real-Space Refinement module of Phenix (41). Volumes and coordinates have been deposited in the PDB with the ID code 6RQJ (42) and the Electron Microscopy Data Bank with ID code EMD-4983 (43). See Table 1 for cryo-EM data collection, refinement, and validation statistics.

### Crystallization, X-Ray Data Collection, and Structure Determination.

CirpT1 was copurified with the C5_MG4 domain by SEC (S75 10/30, GE Healthcare) in PBS and concentrated to 21 mg/mL. The protein complex was with an equal volume of mother liquor containing in 0.02 M Na_2_PO_4_/K_2_PO_4_, 20% (wt/vol) PEG3350, and crystallized in 200-nL drops by a vapor-diffusion method at 21 °C. Crystals were cryoprotected in mother liquor supplemented with 30% glycerol and flash-frozen in liquid N_2_. Data were collected on beamline I03 at the Diamond Light Source (Harwell, United Kingdom), wavelength: 0.9762 Å, as specified in Table 2. The structure of CirpT1-C5_MG4 was solved by molecular replacement using MolRep (44) within CCP4 (45) with the structures of C5–OmCI–RaCI (PDB ID code 5HCC). The structure of CirpT1 was manually built into difference density and the model subjected to multiple rounds of manual rebuilding in Coot (40) and refinement in Phenix (41). The structure of the complex is characterized by the statistics shown in Table 2. Structure factors and coordinates have been deposited in the PDB with the ID code 6RPT (46). Interactions between CirpT1 and C5_MG4 have been predicted by PISA (20). Protein structure ures for both EM and X-ray structures were prepared using Pymol v2.0 (Schrödinger) and ChimeraX (47).

## Supplementary Material

Supplementary File
