# Supplementary material for: An inhibitor of complement C5 provides structural insights into activation
Source: Proc Natl Acad Sci U S A. 2019 Dec 23;117(1):362–70. doi: 10.1073/pnas.1909973116 (PMC6955305; doi:10.1073/pnas.1909973116)
Supplement: Supplementary File [file pnas.1909973116.sapp.pdf]

PNAS
www.pnas.org

Supplementary Information for

A novel inhibitor of complement C5 provides structural insights into activation

Martin P. Reichhardt[1], Steven Johnson[1], Terence Tang[1,b], Thomas Morgan[1,c], Nchimunya Tebeka[1,d,], Niko Popitsch[2,e], Justin C. Deme[1,3], Matthijs M. Jore[1,a] and Susan M. Lea[1,3]

Paste corresponding author name here
Email: susan.lea@path.ox.ac.uk

This PDF file includes:

Figures S1 to S2
Table S1

www.pnas.org/cgi/doi/10.1073/pnas.1909973116

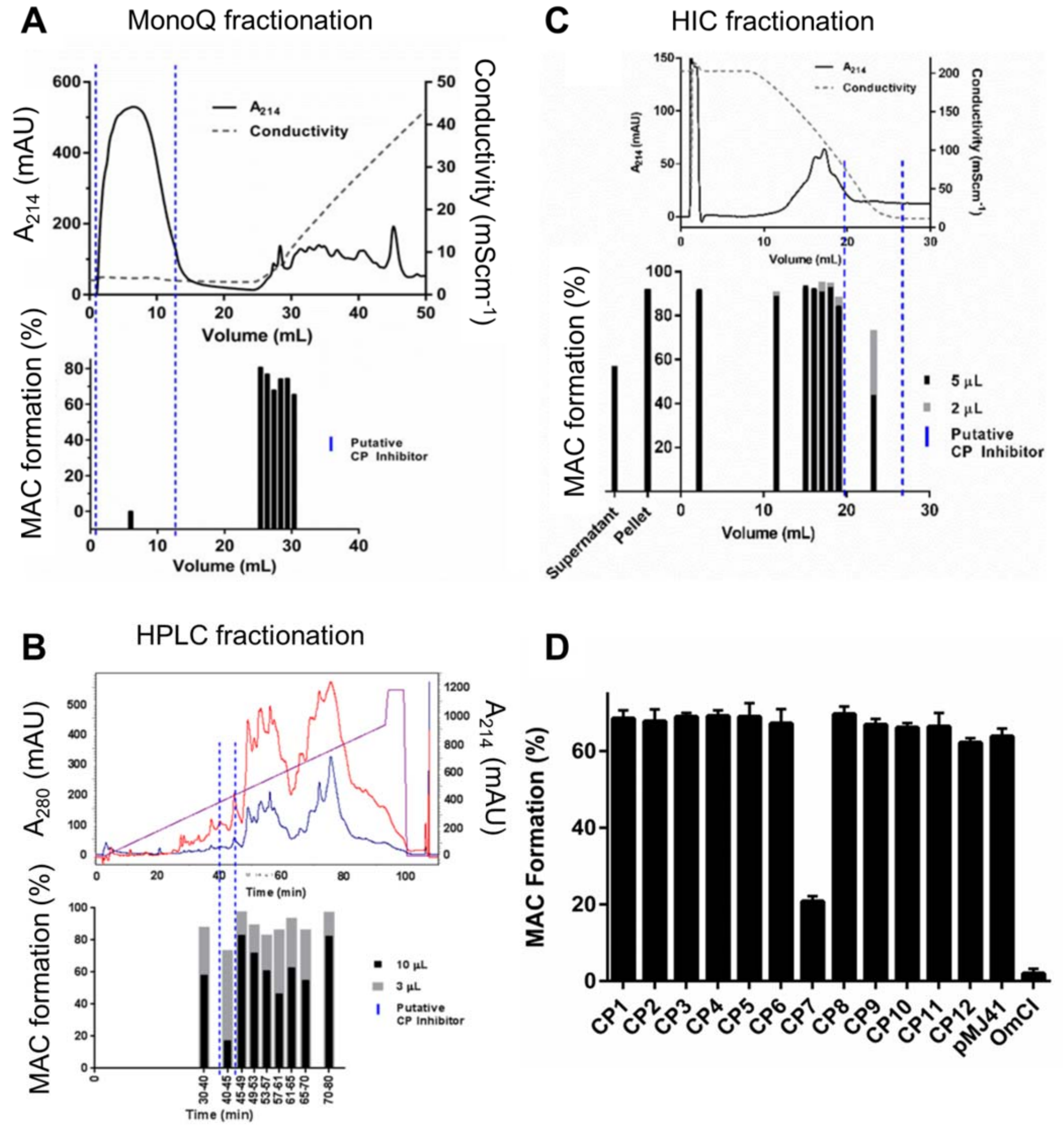

Fig. S1: Fractionation of *Rhipicephalus pulchellus* salivary gland extracts. Proteins in homogenized gland extracts were subjected to sequential chromatographic steps and tested for the ability to inhibit complement. **A)** MonoQ Fractionation: The sample was loaded onto a MonoQ 5/50 GL column (GE), washed and eluted by a 0–0.5 M NaCl. **B)** High-pressure liquid chromatography (HPLC).The active component eluted after 35-40 min. **C)** Hydrophobic Interaction Chromatography (HIC) on a 1 mL HiTrap Butyl HP column (GE), eluted in a 1.7 – 0 M $(NH_4)_2SO_4$ gradient. All fractions were buffer exchanged to PBS, concentrated, and tested for complement inhibition as indicated. **D)** Protein hits from the HIC fraction, obtained by ESI-MS/MS analysis using a novel tick sialome cDNA library, were tested for complement inhibition. CP1=comp1449_seq0,CP2=comp1449_seq1, CP3=comp299_seq17, CP4=comp203_seq0, CP5=comp1933_seq2, CP6=comp282_seq0, CP7=comp53_seq2, CP8=comp65_seq1, CP9=comp3379_seq1, CP10=comp7035_seq0, CP11=comp291_seq1, and CP12=comp22_seq0. OmCI inhibits the terminal pathway of complement and is the positive control. 5 μL of cell culture supernatant was tested by ELISA 8 days after transfection. pMJ41=pExpreS2-2 + N-terminal His_6 tag. Error bars=SEM; n=3.

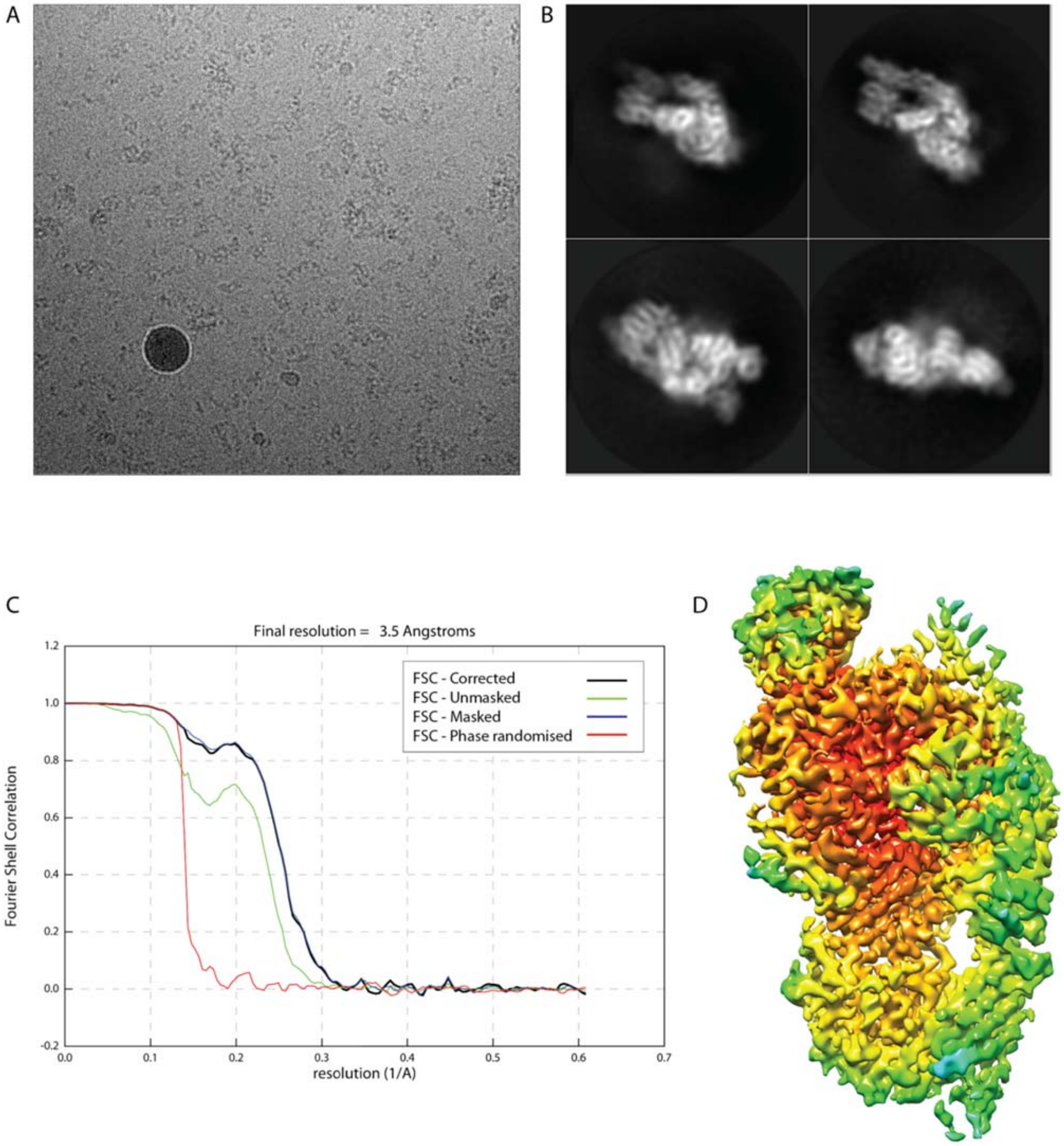

Fig. S2: Single particle cryo-EM of C5-OmCI-RaCI-CirpT1 complex. (A) Representative micrograph. **(B)** Selected reference-free 2D class averages. **(C)** Gold-standard FSC curve of the final map calculated using a soft-edged mask. **(D)** Local resolution estimates of the final map calculated using Relion-LocalRes.

Acc. No.	Re-name	Score	Mature Protein Mass (Da)	pI	BLAST Top Hit	BLAST E score	Blast RefSeq
comp1449_seq0	CP1	167	16482	6.54	None		
comp1449_seq1	CP2	142	17122	6.47	None		
comp299_seq17	CP3	131	13761	9.57	Putative Salivary Secreted Basic Tail Protein	3.00E-06	ACB70313.1
comp203_seq0	CP4	100	24722	8.26	None		
comp1933_seq2	CP5	97	17139	8.26	Rhipilin	1.00E-22	AFN22082.1
comp282_seq0	CP6	96	7922	8.20	None		
comp53_seq2	CP7	89	9898	5.44	Hypothetical protein (*Ixodes scapularis*)	8.00E-04	AAY66509.1
comp65_seq1	CP8	61	11876	8.67	Kielin/chordin-like protein	0.2	XP_012261665.1
comp3379_seq1	CP9	57	21166	8.96	Rhipilin-2	2.00E-29	AFN22082.1
comp7035_seq0	CP10	56	6457	8.24	None		
comp291_seq1	CP11	53	6366	7.79	None		
comp22_seq0	CP12	14	15750	8.64	None		

Table S1: CP Candidate Inhibitor proteins identified by ESI-MS/MS analysis
